# Supplementary material for: Deficit Irrigation at Pre-Anthesis Can Balance Wheat Yield and Water Use Efficiency under Future Climate Change in North China Plain
Source: Biology (Basel). 2022 Apr 30;11(5):692. doi: 10.3390/biology11050692 (PMC9138343; doi:10.3390/biology11050692)
Supplement: Supplementary file 1 [file biology-11-00692-s001.zip › biology-1679414-supplementary.pdf]

Table S1. Detailed agronomic management options used in this study.

**Numbers**	**Treatments**	**Water deficit**			**N fertilizer rates (kg N ha^{-1})**	**Numbers**	**Treatments**	**Water deficit**			**N fertilizer rates (kg N ha^{-1})**
		**Sowing to flowering**	**Flowering to grain filling**	**Grain filling to maturity**				**Sowing to flowering**	**Flowering to grain filling**	**Grain filling to maturity**	
**1**	**DI1_N0**	**≤0.2PAWC**	**≤0.75 PAWC**	**≤0.65PAWC**	0	33	DI5_N4	≤0.6PAWC	≤0.75 PAWC	≤0.65PAWC	200
2	DI1_N1	≤0.2PAWC	≤0.75 PAWC	≤0.65PAWC	50	34	DI5_N5	≤0.6PAWC	≤0.75 PAWC	≤0.65PAWC	250
3	DI1_N2	≤0.2PAWC	≤0.75 PAWC	≤0.65PAWC	100	35	DI5_N6	≤0.6PAWC	≤0.75 PAWC	≤0.65PAWC	300
4	DI1_N3	≤0.2PAWC	≤0.75 PAWC	≤0.65PAWC	150	36	DI6_N0	≤0.7PAWC	≤0.75 PAWC	≤0.65PAWC	0
5	DI1_N4	≤0.2PAWC	≤0.75 PAWC	≤0.65PAWC	200	37	DI6_N1	≤0.7PAWC	≤0.75 PAWC	≤0.65PAWC	50
6	DI1_N5	≤0.2PAWC	≤0.75 PAWC	≤0.65PAWC	250	38	DI6_N2	≤0.7PAWC	≤0.75 PAWC	≤0.65PAWC	100
7	DI1_N6	≤0.2PAWC	≤0.75 PAWC	≤0.65PAWC	300	39	DI6_N3	≤0.7PAWC	≤0.75 PAWC	≤0.65PAWC	150
8	DI2_N0	≤0.3PAWC	≤0.75 PAWC	≤0.65PAWC	0	40	DI6_N4	≤0.7PAWC	≤0.75 PAWC	≤0.65PAWC	200
9	DI2_N1	≤0.3PAWC	≤0.75 PAWC	≤0.65PAWC	50	41	DI6_N5	≤0.7PAWC	≤0.75 PAWC	≤0.65PAWC	250
10	DI2_N2	≤0.3PAWC	≤0.75 PAWC	≤0.65PAWC	100	42	DI6_N6	≤0.7PAWC	≤0.75 PAWC	≤0.65PAWC	300
11	DI2_N3	≤0.3PAWC	≤0.75 PAWC	≤0.65PAWC	150	43	DI7_N0	≤0.8PAWC	≤0.75 PAWC	≤0.65PAWC	0
12	DI2_N4	≤0.3PAWC	≤0.75 PAWC	≤0.65PAWC	200	44	DI7_N1	≤0.8PAWC	≤0.75 PAWC	≤0.65PAWC	50
13	DI2_N5	≤0.3PAWC	≤0.75 PAWC	≤0.65PAWC	250	45	DI7_N2	≤0.8PAWC	≤0.75 PAWC	≤0.65PAWC	100
14	DI2_N6	≤0.3PAWC	≤0.75 PAWC	≤0.65PAWC	300	46	DI7_N3	≤0.8PAWC	≤0.75 PAWC	≤0.65PAWC	150
15	DI3_N0	≤0.4PAWC	≤0.75 PAWC	≤0.65PAWC	0	47	DI7_N4	≤0.8PAWC	≤0.75 PAWC	≤0.65PAWC	200
16	DI3_N1	≤0.4PAWC	≤0.75 PAWC	≤0.65PAWC	50	48	DI7_N5	≤0.8PAWC	≤0.75 PAWC	≤0.65PAWC	250
17	DI3_N2	≤0.4PAWC	≤0.75 PAWC	≤0.65PAWC	100	49	DI7_N6	≤0.8PAWC	≤0.75 PAWC	≤0.65PAWC	300
18	DI3_N3	≤0.4PAWC	≤0.75 PAWC	≤0.65PAWC	150	50	FI_N0	≤0.8PAWC	≤0.8PAWC	≤0.8PAWC	0
19	DI3_N4	≤0.4PAWC	≤0.75 PAWC	≤0.65PAWC	200	51	FI_N1	≤0.8PAWC	≤0.8PAWC	≤0.8PAWC	50
20	DI3_N5	≤0.4PAWC	≤0.75 PAWC	≤0.65PAWC	250	52	FI_N2	≤0.8PAWC	≤0.8PAWC	≤0.8PAWC	100
21	DI3_N6	≤0.4PAWC	≤0.75 PAWC	≤0.65PAWC	300	53	FI_N3	≤0.8PAWC	≤0.8PAWC	≤0.8PAWC	150
22	DI4_N0	≤0.5PAWC	≤0.75 PAWC	≤0.65PAWC	0	54	FI_N4	≤0.8PAWC	≤0.8PAWC	≤0.8PAWC	200
23	DI4_N1	≤0.5PAWC	≤0.75 PAWC	≤0.65PAWC	50	55	FI_N5	≤0.8PAWC	≤0.8PAWC	≤0.8PAWC	250
24	DI4_N2	≤0.5PAWC	≤0.75 PAWC	≤0.65PAWC	100	56	FI_N6	≤0.8PAWC	≤0.8PAWC	≤0.8PAWC	300
25	DI4_N3	≤0.5PAWC	≤0.75 PAWC	≤0.65PAWC	150	57	RN_N0		Rainfed		0
26	DI4_N4	≤0.5PAWC	≤0.75 PAWC	≤0.65PAWC	200	58	RN_N1		Rainfed		50
27	DI4_N6	≤0.5PAWC	≤0.75 PAWC	≤0.65PAWC	250	59	RN_N2		Rainfed		100
28	DI4_N7	≤0.5PAWC	≤0.75 PAWC	≤0.65PAWC	300	60	RN_N3		Rainfed		150
29	DI5_N0	≤0.6PAWC	≤0.75 PAWC	≤0.65PAWC	0	61	RN_N4		Rainfed		200
30	DI5_N1	≤0.6PAWC	≤0.75 PAWC	≤0.65PAWC	50	62	RN_N5		Rainfed		250
31	DI5_N2	≤0.6PAWC	≤0.75 PAWC	≤0.65PAWC	100	63	RN_N6		Rainfed		300
32	DI5_N3	≤0.6PAWC	≤0.75 PAWC	≤0.65PAWC	150						

Note: PAWC is plant available water holding capacity in 0–2m soil layer.

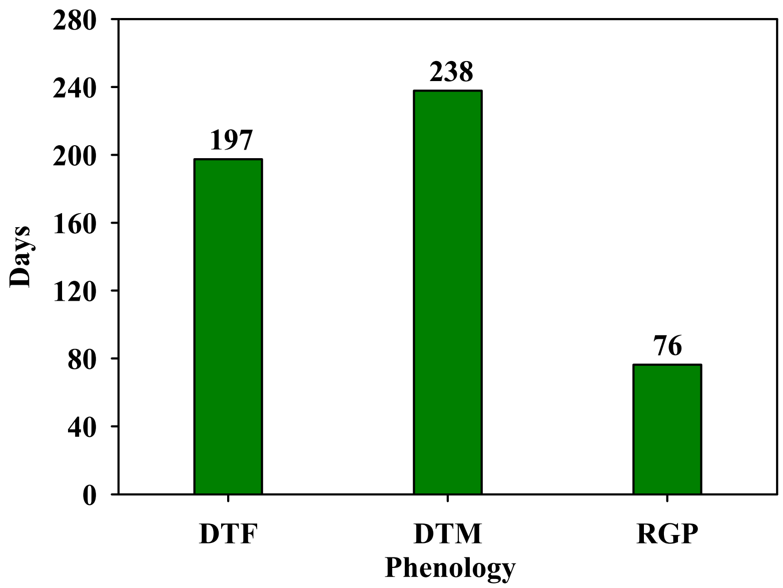

Figure S1. Simulated days from sowing to flowering (DTF), days from sowing to maturity (DTM) and reproduction growth period (RGP) in the baseline (1961–2000).

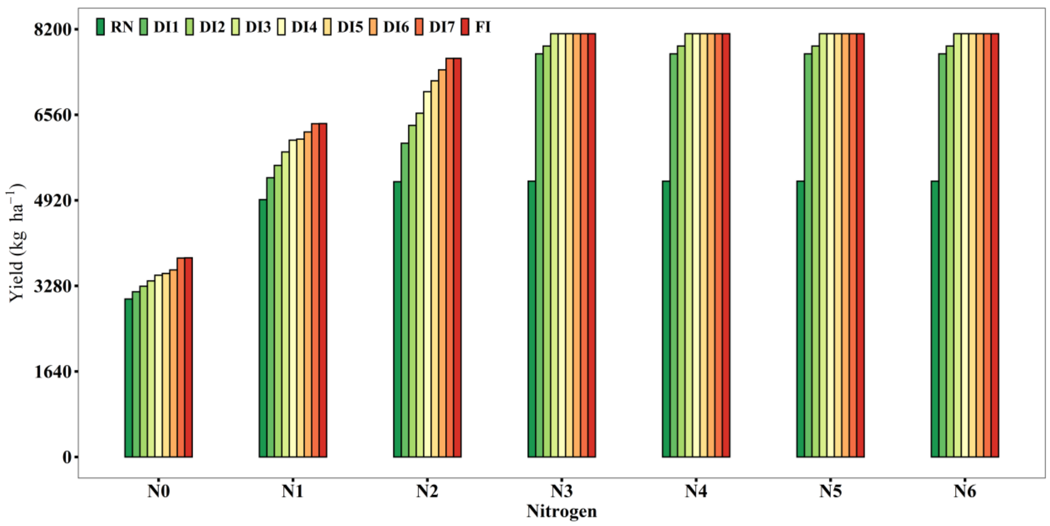

Figure S2. Simulated average yield under nine irrigation treatments across seven N fertilizer rates in the baseline (1961–2000).

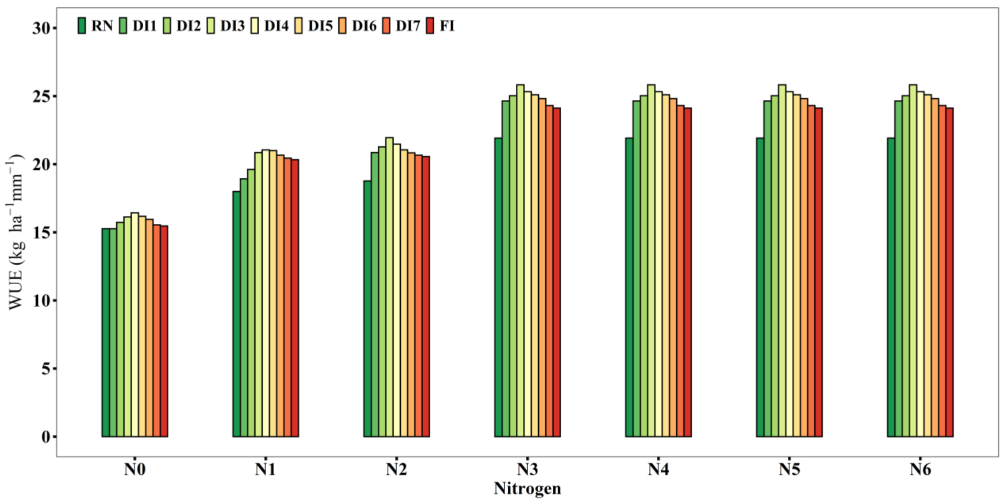

Figure S3. Simulated average WUE under nine irrigation treatments across seven N fertilizer rates in the baseline (1961−2000).

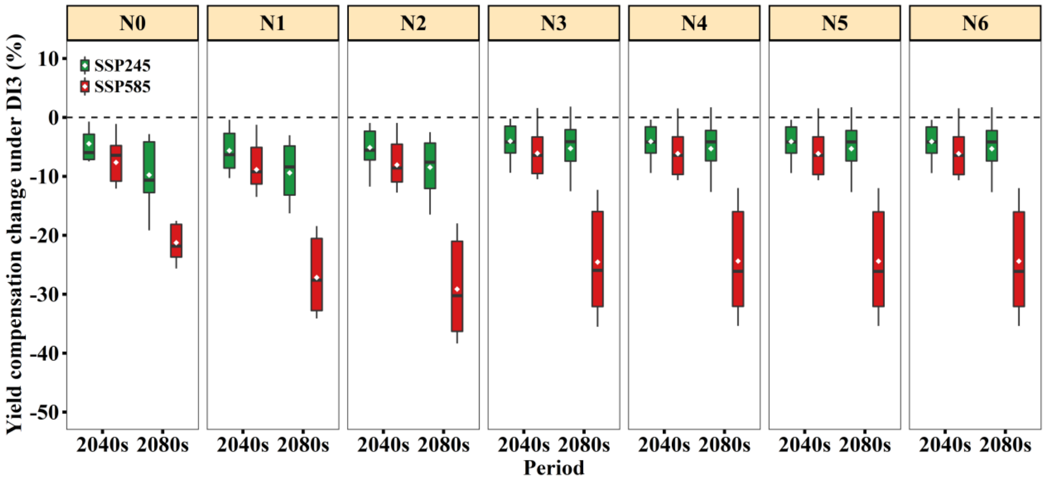

Figure S4. The change of compensation effect on yield under DI3 in 2040s and 2080s under SSP245 and SSP585 based on 21 GCMs across seven N fertilizer rates relative to baseline. Box boundaries indicate the 25th and 75th percentiles across 21 GCMs, and whiskers below and above the box denote as the 10th and 90th percentiles. The black lines and white dots inside the box indicate the median and mean, respectively.

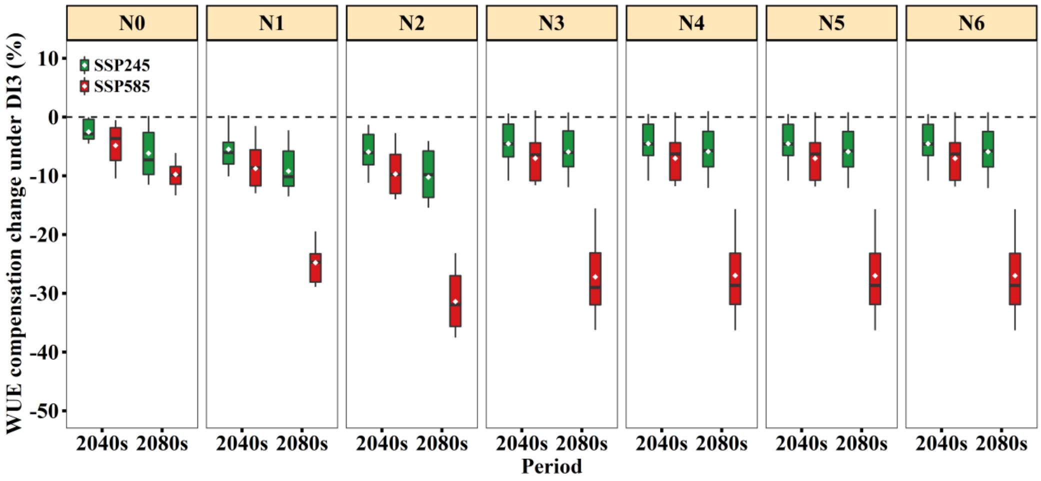

Figure S5. The change of compensation effect on WUE under DI3 in 2040s and 2080s under SSP245 and SSP585 based on 21 GCMs across seven N fertilizer rates relative to baseline. Box boundaries indicate the 25th and 75th percentiles across 21 GCMs, and whiskers below and above the box denote as the 10th and 90th percentiles. The black lines and white dots inside the box indicate the median and mean, respectively.
